# Kinetics of Amyloid Aggregation: A Study of the GNNQQNY Prion Sequence

**DOI:** 10.1371/journal.pcbi.1002782

**Published:** 2012-11-29

**Authors:** Jessica Nasica-Labouze, Normand Mousseau

**Affiliations:** Département de Physique and GÉPROM, Université de Montréal, Montréal, Québec, Canada; University of Maryland, Baltimore, United States of America

## Abstract

The small amyloid-forming GNNQQNY fragment of the prion sequence has been the subject of extensive experimental and numerical studies over the last few years. Using unbiased molecular dynamics with the OPEP coarse-grained potential, we focus here on the onset of aggregation in a 20-mer system. With a total of 16.9 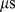 of simulations at 280 K and 300 K, we show that the GNNQQNY aggregation follows the classical nucleation theory (CNT) in that the number of monomers in the aggregate is a very reliable descriptor of aggregation. We find that the critical nucleus size in this finite-size system is between 4 and 5 monomers at 280 K and 5 and 6 at 300 K, in overall agreement with experiment. The kinetics of growth cannot be fully accounted for by the CNT, however. For example, we observe considerable rearrangements after the nucleus is formed, as the system attempts to optimize its organization. We also clearly identify two large families of structures that are selected at the onset of aggregation demonstrating the presence of well-defined polymorphism, a signature of amyloid growth, already in the 20-mer aggregate.

## Author Summary

Protein aggregation plays an important pathological role in numerous neurodegenerative diseases such as Alzheimer's, Parkinson's, Creutzfeldt-Jakob, the Prion disease and diabetes mellitus. In most cases, misfolded proteins are involved and aggregate irreversibly to form highly ordered insoluble macrostructures, called amyloid fibrils, which deposit in the brain. Studies have revealed that all proteins are capable of forming amyloid fibrils that all share common structural features and therefore aggregation mechanisms. The toxicity of amyloid aggregates is however not attributed to the fibrils themselves but rather to smaller more disordered aggregates, oligomers, forming parallel to or prior to fibrils. Understanding the assembly process of these amyloid oligomers is key to understanding their toxicity mechanism in order to devise a possible treatment strategy targeting these toxic aggregates. Our approach here is to computationally study the aggregation dynamics of a 20-mer of an amyloid peptide GNNQQNY from a prion protein. Our findings suggest that the assembly is a spontaneous process that can be described as a complex nucleation and growth mechanism and which can lead to two classes of morphologies for the aggregates, one of which resembles a protofibril-like structure. Such numerical studies are crucial to understanding the details of fast biological processes and complement well experimental studies.

## Introduction

The aggregation of misfolded amyloid proteins into fibrils is a hallmark of many neurodegenerative diseases such as Alzheimer's, Parkinson's, and Huntington's diseases [1]–[5] and understanding amyloid aggregation mechanisms is crucial for controlling their destructive consequences. Fibrils are known to be ordered insoluble assemblies with a core cross-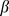 structure. They are not the only aggregated species involved, however, and oligomers, smaller intermediates on or off the fibril formation pathway, have been found to be responsible for amyloid cytotoxicity [6]–[8]. Their role in amyloid aggregation is still a matter of debate but significant efforts have gone into better understanding and characterizing their structure and dynamics both experimentally [9]–[11] and computationally [12]–[17]. Oligomers are often found to be precursors to amyloid fibrils. They could also, in some cases, appear as the product of a competition between the ordered fibrillar and amorphous globular morphologies, forming via different assembly pathways. This widespread characteristic of amyloid proteins is described as polymorphism [18]–[20] and is under kinetic control [21]. The presence of oligomers is therefore crucial for the fibrillisation process as well as the final morphology of fibrils [22] and understanding their kinetics of formation could be the key to controlling this polymorphism.

The aggregation of amyloid proteins is a highly cooperative self-assembly mechanism, which is often described as a complex nucleation and growth process [23]. The nucleation step, in a supersaturation environment, consists of a series of stochastic events leading to the formation of metastable seeds for the oligomer or fibril to grow on [24]. Nucleation kinetics display two characteristic properties: the presence of 1) a lag time before aggregates can be detected and 2) a maximum growth rate after nucleation is triggered [25], [26]. Direct experimental observations of nucleation and growth have been reported [27]–[30] and nucleation was always found to be the rate-limiting step of amyloid formation [26].

The aim of the present work is to investigate the dynamics of amyloid aggregation and the forces driving self-assembly for the 20-mer system of the amyloidogenic GNNQQNY peptide using molecular dynamics (MD) and a coarse-grained potential (OPEP). The nucleation specificity of the N-terminal region (9–39) of the budding yeast prion protein Sup35, GNNQQNY, is well understood. This small heptapeptide alone drives the entire Sup35 protein to self-assemble into amyloid fibrils [31] and, when isolated, displays the same amyloid properties and aggregation kinetics as the full-length Sup35 protein [32]. In addition, its cross-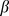 spine structure has been determined at the atomic level by X-ray crystallography [33]. It is therefore a very good candidate to the study of amyloid aggregation kinetics and numerous computations have been performed on the GNNQQNY sequence to characterize the onset of aggregation for this model [34]–[38]. This work expands on our previous multi-scale thermodynamic study of different sizes of GNNQQNY systems, where we identified the morphologies accessible to the trimer, dodecamer and 20-mer [39]. Now, we focus on the aggregation kinetics using long MD simulations of unbiased spontaneous self-assembly. We offer a full analysis of the onset of aggregation for GNNQQNY peptides at a refined coarse-grained level. A total of 16.9 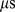 of simulations have been collected to allow statistically relevant analyses. Altogether, our results indicate the presence of a nucleated-polymerization process intertwined with oligomer-involving mechanisms, thus leading to a certain degree of polymorphism that is already clearly established for the 20-mer.

## Materials and Methods

Following Ref. [39], which showed that the GNNQQNY amyloid aggregates generated with the coarse-grained OPEP forcefield [40] were reasonably preserved in long explicit solvent all-atom MD simulations, we revisit this system focusing, this time, on the kinetics of the aggregation process.

### System description

As in our previous study, we perform implicit solvent coarse-grained molecular dynamics (MD) simulations using the OPEP potential version 3.0 [40]. OPEP is designed for efficient protein folding and structure prediction of large systems over long timescales and is also accurate for studying thermodynamics [41]. In OPEP, all heavy backbone atoms are fully represented (N, H, 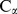, C and O). Side chains, for their part, are reduced to a single bead with appropriate geometrical properties and van der Waals radius. The implicit effects of the solvent are included in the interaction parameters of the potential energy function, as detailed elsewhere [40], [42]. OPEP is a well tested potential and has been implemented with a palette of numerical methods such as Monte-Carlo [42]–[46], the activation-relaxation technique (ART nouveau) [47]–[52], MD [41], [53]–[55] and REMD [39], [56]–[59].

Here, two sets of single temperature MD are performed on a 20-mer of GNNQQNY, with both terminii of each peptide charged, in order to characterize in details the kinetics of aggregation. The first set consists of a total of 152 100 ns simulations (76 at 280 K and 76 at 300 K) with configurations saved every 5000 steps. The choice of temperatures is motivated by the fact that 280 K and 300 K are temperatures below and above the transition temperature previously found for the 20-mer of GNNQQNY. As explained below, the initial atomic positions taken for this set are extracted from the simulations reported in Ref. [39]. An additional 10 30 ns simulations are then carried out from a subset of the starting atomic positions of the previous simulation set (5 at 280 K and 5 at 300 K) with configurations saved every 50 steps to better monitor the detailed evolution of the system during the nucleation phase. All simulations are independent, starting with different random Boltzmann distributed velocities. In every case, we maintain simulation conditions as close as those of Ref. [39], with a Berendsen thermostat for temperature control [60], an integration time step of 1.5 fs and periodic boundary conditions applied to a box 200 Å in size containing 20 monomers of GNNQQNY, which represents a constant 4.15 mM concentration.

**Figure 1:**
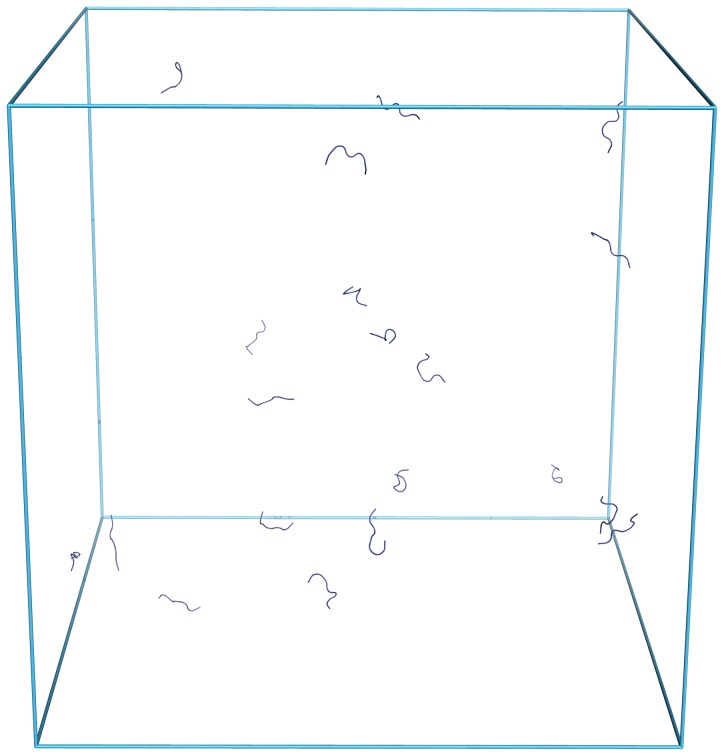
Typical starting structure for our MD simulations. The 20 peptides are initially in a random coil conformation and placed in a 200 Å box to ensure a 4.15 mM concentration.

For simplicity, the starting random structures for our simulations were extracted from the high-temperature set generated in our previous REMD OPEP runs of the GNNQQNY 20-mer [39]. A typical starting structure for our simulations is shown in Figure 1 with all 20 peptides isolated and in random coil conformations. At the start of each run, a minimization procedure is performed using a combination of the steepest descent algorithm and the conjugate gradient method [61], followed by a thermalization of 50 000 steps (0.075 ns) to ensure that all conformations are fully thermalized.

Because of the implicit solvent treatment as well as the peptide's coarse-grained representation, that decrease the number of degrees of freedom, the aggregation kinetics is accelerated. It is therefore not possible to establish a direct connection between the aggregation time observed in the simulation and in experiments. However, as shown in Ref. [41], the thermodynamical properties are, at least qualitatively, maintained. The simulations presented here, therefore, should provide the right qualitative picture for the first steps in the kinetics of aggregation.

### Analysis

Most of the analysis on the nucleation and growth kinetics is carried out using a clustering tool [39] adapted to multiprotein assembly and designed to classify 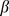-sheet clusters based on strand attachment. For the purpose of this work, this procedure can also handle the calculation of kinetic association and dissociation rates. To assess strand attachment, the criterion used to define and calculate hydrogen bonds between strands is similar to the DSSP definition [62]. A peptide belongs to a cluster if it is attached to another strand of that cluster by at least two hydrogen bonds. An additional criterion is applied on dihedrals 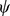 and 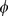 angles to determine if a given strand in a cluster has enough amino acids in 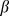-conformation. For each amino acid the 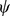 and 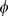 angles are calculated and if they satisfy the region 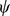
(in degrees): [−180∶−150;0∶180], 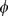 (in degrees): [−180∶0;150∶180] (corresponding approximately to the 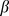 region of the Ramachandran plot [63]), the amino acid is in a 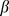 state. A GNNQQNY peptide is considered in a 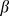 state if at least three of its residues are in the 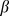 region. If a peptide is not found to be in a 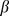 state, it is excluded from the cluster. This determination of secondary structure is solely used to determine cluster membership of the strands. The clustering analysis allows us to measure accurately the evolution of clusters over time based on local information and to monitor their properties such as the orientation of strands within 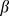-sheets (i.e., parallel or anti-parallel). For purposes other than cluster determination, secondary structure calculations are made using the STRIDE program [64].

In order to look at the aggregation process in more details, we also consider the association and dissociation rates of the clusters in the following way. With 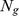 the concentration of 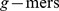, we consider aggregation as a dynamical process involving both association and dissociation that can occur either one monomer or more than one monomer at a time. The former is referred to as growth by monomer addition/monomer loss while the latter is described as being a mix of two processes, oligomer fusion/fragmentation and formation/destruction of oligomers from/into monomers, when involving more than one monomer at a time. We can then define the net rate of creation of 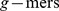 as
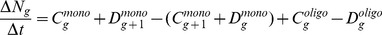
(1)where 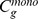 and 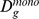 are the creation rate of 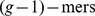 into 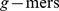 and the destruction rate of 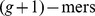 into 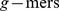, 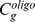 and 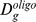 are the creation and destruction rates of 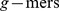 either directly from/into monomers, or from the fusion/fragmentation of other sizes of oligomers. All the C and D rates are calculated from our clustering tool and allow us to gather statistics on the microscopic kinetic events and mechanisms.

## Results/Discussion

We present a study of the aggregation kinetics of 20-mer GNNQQNY oligomers under a 4.15 mM concentration, the same concentration that was used in our previous multiscale thermodynamic study of the GNNQQNY 20-mer system [39].

We first present the general results obtained from the 100 ns MD simulations whose configurations were saved every 7.5 ps (5000 simulation steps) with an initial configuration selected as discussed in the materials and methods section. Then we discuss results from the 30-ns MD simulations whose configurations are saved every 75 fs (50 simulation steps) to better study the detailed association and dissociation kinetics of oligomers.

### Observed kinetics

**Figure 2:**
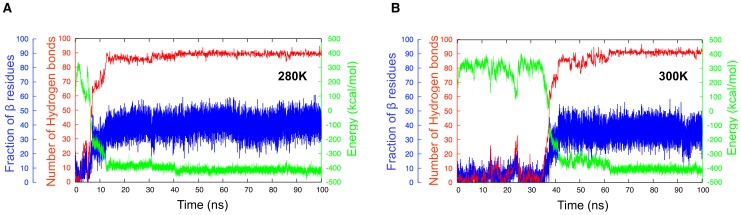
Time evolution of the structural properties of the GNNQQNY 20-mer. The three curves represent the total potential energy (in green), the total number of hydrogen bonds (in red) and the secondary structure (in blue) for a typical 100 ns simulation at (a) 280 K and (b) 300 K.

At the lowest temperature of 280 K, all 76 100 ns simulations lead to ordered amyloid oligomers formation. In all cases, aggregation is accompanied by a sudden drop of the total potential energy of the system, by over 600 kcal/mol over less than 10 ns, and by an increase in the 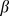-sheet content of 30%, as calculated with the STRIDE program [64]. While the exact energy value is not significative, due to the implicit-solvent coarse-grained nature of our energy model, its drop corresponds to the formation of a more stable structure. The system then stays in a minimum of energy and both the number of hydrogen bonds and the amount of secondary structure stabilize. As shown in Figure 2(a), which presents a typical aggregation run, the 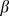-content in the structures fluctuates typically around 50%, near its maximum of 60%, as the glycines and tyrosines end residues of each 20 peptides do not get involved in the 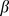-sheet hydrogen bonding. Figure 2(a) also shows the high correlation between the energy drop and the increase in the number of hydrogen bonds as a function of time, suggesting that the cooperativity between hydrogen bonds plays a crucial role in lowering the energy and stabilizing the system.

Aggregation is slower at 300 K and only 68% of the 76 100 ns simulations lead to ordered amyloids. However, as shown in the typical aggregation run in Figure 2(b), the overall ordering follows a trend very similar to that at the lower temperature : a sudden potential energy drop of over 600 kcal/mol over less than 10 ns accompanied by correlated raises in both the number of hydrogen bonds and the 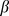-sheet content. If the final number of hydrogen bonds is very similar to that at 280 K, the secondary structure is less stable and tends to fluctuate around 40% rather than 50%.

**Figure 3:**
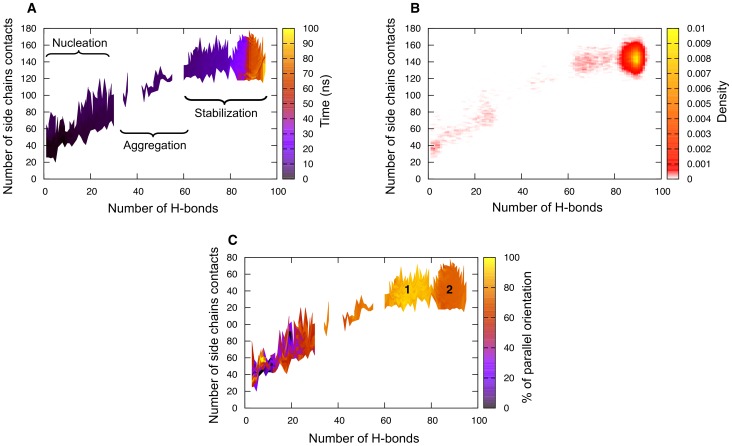
Evolution of the structural properties for the GNNQQNY 20-mer at 280 K as a function of the number of hydrogen bonds and of the number of side chain contacts. (a) Time evolution map of the system. Black regions indicate the beginning of the simulation while yellow regions indicate the end. (b) Density map representing the probability of having a configuration lie in a specific region. Yellow is the highest density and red the lowest. (c) Proportion of parallel 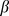-strands. Yellow regions indicate that 100% of the strands are in parallel orientation while black regions indicate that none of the strands are in parallel orientation thus meaning that they all are in antiparallel orientation. Since cluster determination is based on the presence of hydrogen bonds, the percentage of antiparallel orientation of the strands is equal to 1 minus the percentage of parallel orientation. Regions 1 and 2 indicate the two regions of highest parallel orientation. The discontinuities in the maps (a) and (c) is a plotting artifact in low-density regions where the system is rapidly changing during aggregation and there are therefore not enough points to fill the map regions.

In order to describe the assembly process we represent the time evolution, the probability density and the orientation of strands in structures as a function of the number of hydrogen bonds and of the number of contacts between side chains as these two coordinates are the least correlated and are the best measure of how ordered the structures are. Figures 3 and S1 show these quantities for the trajectories plotted in Figure 2 at 280 K and 300 K, respectively.

At 280 K, we observe three distinct kinetic stages over the course of a typical simulation (Figure 3(a)). The first phase is characterized as the nucleation phase, which lasts about 5 ns after the start of the simulation and leads to the formation of the metastable critical nucleus. During this phase, small oligomers form and break under stochastic collisions of the monomers. Seeds below the nucleus size fluctuate considerably, forming and disassembling at a high rate, forming a quasi-equilibrium perfectly reversible process. Once the metastable nucleus forms, the system can move into the aggregation (or growth) phase with a 50% probability, by definition. In this dynamical phase, almost all of the monomers rapidly assemble around the nucleus to form a partially disordered globular oligomer. In general, this stage is very rapid and typically lasts less than 10 ns. During the third phase, which extends over a timescale of up to 80 ns, the aggregate rearranges itself as monomers explore their local configuration environment within the confines of the oligomers, optimizing the energy and, as a consequence, the secondary structure and the number of side chain – side chain contacts (see the last 75 ns in Figure 3 (a) and (c)). This process, which we describe as a stabilization phase, is the slowest of the three and accounts for the dense region in Figure 3(b).

This aggregation process is consistent with the “condensation-ordering” mechanism previously observed experimentally [65] and computationally [12]–[14], [66]. An interesting feature of the kinetics at 280 K is the increase and later dominance of parallel orientation in the structures over time during both the growth and stabilization phases while the structures are mostly antiparallel during the nucleation phase (Figure 3(c)). By looking at the color coding on the right axis, it appears as though the system is loosing some parallel orientation between region 1 and 2 from almost 100% to 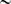
80%. Instead our results indicate that the system continues to evolve and gain some secondary structure between region 1 and 2 of the graph. It is the newly formed 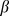-strands that adopt an antiparallel orientation while the parallel content formed during the growth process remains unchanged. As a whole, 91% of the MD simulations at 280 K lead to a final assembly dominated by parallel 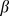-sheets, in agreement with recent experimental findings [33], [67], [68] and computational studies [35], [36], [39], [69], [70].

At 300 K the kinetics globally display the same three phases for nucleation, growth and stabilization of oligomers observed at 280 K, and 95% of the final aggregated structures display a dominance of parallel orientation of the 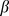-strands (Figure S1). The main difference between the two temperatures (Figure 2) is in the lag time associated with the nucleation phase: while the average lag time is found to be 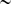
13 ns at 280 K, it increased to 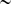
56 ns at 300 K, leading to a denser nucleation region on the probability map (Figure S1(b)). Mechanistically, this increase in nucleation time can be explained by the presence of bigger thermal fluctuations that destabilize the metastable aggregates, preventing nucleation.

**Figure 4:**
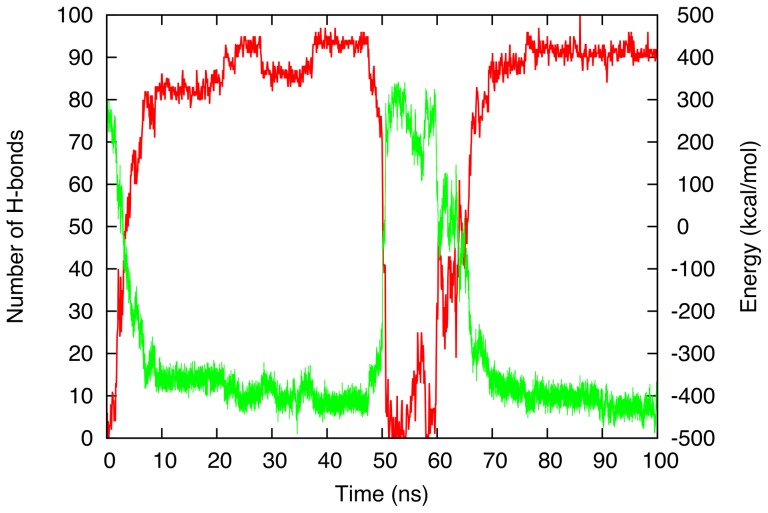
Time evolution of the structural properties of a GNNQQNY 20-mer simulation that shows reversibility in the formation of a structured aggregate. The two curves represent the total potential energy (in green) and the total number of hydrogen bonds (in red).

While most simulations at 280 K and 300 K generate a single aggregation event, we observe reversibility for 34% of aggregation events at 280 K against 40% at 300 K. In these cases, such as in the example shown in Figure 4, monomers undergo a complete aggregation process up to and including the stabilization phase before the reverse reaction takes place, leading to a completely or partially random structure. For some simulations, this reversible transition was even observed to occur a few times during the 100 ns run. The presence of reversibility tells us that even though the free energy barrier for forming a 20-mer oligomer is high, the system is not completely biased towards the formation of an ordered oligomer. Thermal fluctuations for this 20-mer are sufficient to destabilize ordered oligomers on a relatively short time scale, a process that cannot be achieved in all coarse-grained aggregation simulations [16], [71] but which is crucial in order to describe aggregation kinetics correctly.

#### Diversity of the trajectories

**Figure 5:**
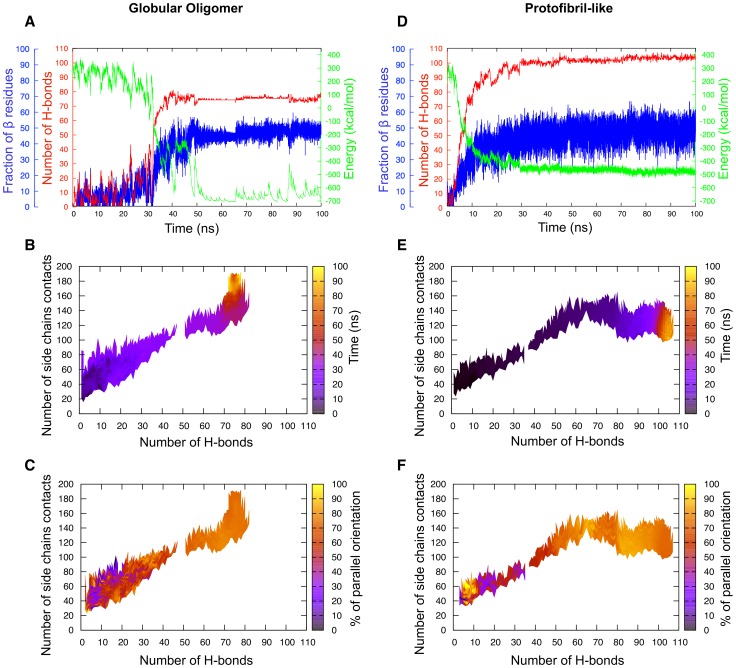
Competition between the globular oligomer (R1) and the protofibril (R2) at 280 K. (a) Low energy profile displaying a low amount of hydrogen bonds for the globular oligomer; (b) corresponding time map as a function of the number of hydrogen bonds and of the side chain contacts; (c) corresponding amount of parallel 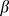-strands as a function of the same parameters; (d) kinetic profile displaying a particularly high number of hydrogen bonds in the protofibril structure; (e) corresponding time map as a function of the number of hydrogen bonds and of the side chain contacts. f) corresponding amount of parallel beta-strands as a function of the same parameters. These graphs demonstrate the existence of a competition between the globular structure with a low amount of hydrogen bonds and a high amount of contacts and the protofibril structure with a high amount of hydrogen bonds and a low amount of contacts. The actual structures are shown in Figure 6 (a) and (b).

**Figure 6:**
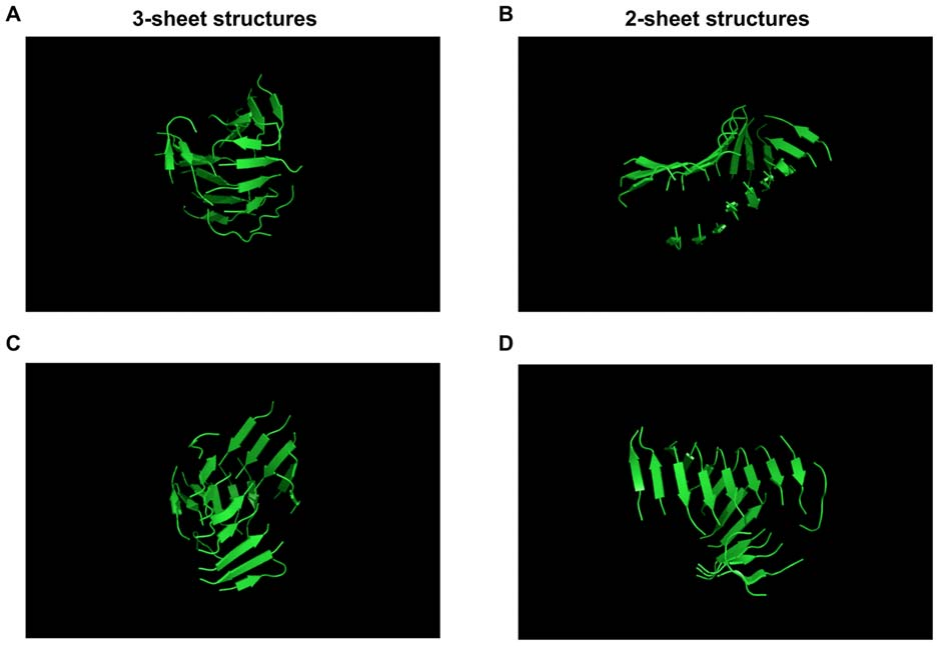
Diversity of the morphologies. (a) Oligomer displaying an extremely high amount of contacts (simulation R1). (b) Protofibril-like structure displaying a high number of hydrogen bonds (simulation R2); (c) typical 3-plus sheet structure often generated in our simulations; (d) typical 2-sheet structure.

Among the 76 simulations at 280 K, we find two simulations that display extreme behavior (Figure 5). The first one, R1, (panels (a) to (c)) assembles into a very low-energy structure, reaching as low as 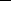
750 kcal/mol in places (lower than the minimum energy of 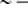
450 kcal/mol for a typical aggregation process at 280 K) (Figure 5(a)). Unexpectedly, this low energy is associated with the presence of a relatively small number of hydrogen bonds, between 70 to 80 at maximum, while it is usually between 90 to 100 (see Figure 3). This low number of hydrogen bonds is compensated by a very high number of side chain – side chain contacts that reaches 190 and more, well above the usual maximum of 170 (see the yellow section of Figure 5(b)). In Figure 5(a) the maximum amount of secondary structure is 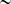
45%, lower than a typical structure in our simulations and seems to fluctuate significantly less than for a typical structure, due to the small number of hydrogen bonds in the structure. The second extreme simulation, R2, (panels (d) to (f) of Figure 5), shows the opposite behavior, with a high number of hydrogen bonds (panel (d)) and a very low number of contacts (between 100 and 120) during the last 20 ns (in yellow in Figure 5(f)). The secondary structure here behaves similarly to a typical simulation and fluctuates around 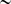
50% (Figure 5(d)). Looking at the final morphologies of the structures in each case (Figure 6 (a) and (b)), we see that the first simulation, with a high number of side chain – side chain contacts and fewer hydrogen bonds, leads to a very compact oligomer composed of several small 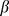-sheets while the second simulation, with a large number of hydrogen bonds and fewer contacts, favors a protofibril-like structure made of two long twisted 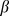-sheets facing each other.

These two simulations demonstrate the existence of a competition between the optimization of the number of contacts and hydrogen bonds. This competition generates a family of formation pathways that can lead to formally different topologies, ranging from a compact globular oligomer to an extended protofibril-like structure [10], [39], [72], [73]. In all cases, the final structures display a very high parallel 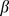-content (Figure 5 (c) and (f)). By comparing these two extreme cases to morphologies of typical simulations at 280 K, we note that, statistically, most of these fall squarely into one of two general morphologies: a class of 3-plus sheet structures that seem rather compact (Figure 6(c)), which resembles the compact oligomer, and a class of 2-sheet structures (panel (d)) similar to the protofibril-like structure (Figure 6 (b)) but with a distribution of orientations between the two 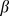-sheets. The two extreme structures, which we had already observed in our previous study [39], can therefore be considered as the optimal cases of the two large structure families of ordered amyloid aggregates generated in our simulations.

### Details of the aggregation kinetics - the “growth” phase

**Figure 7:**
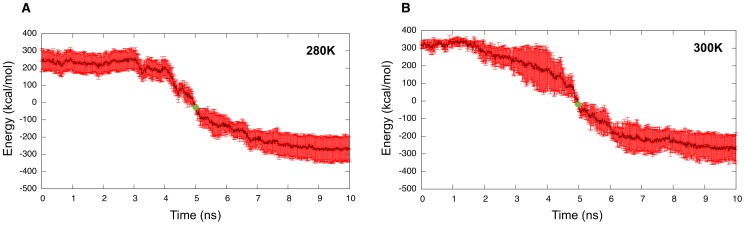
Detailed kinetics of 30 ns MD simulations. a) Average energy profile at 280 K (5 sets of data were used). The average curve is shown in black while the standard deviation is shown in red. The green dot represents the point around which the profiles were centered before computing the statistics over a 10 ns window (5 ns before and 5 ns after); b) Average energy profile at 300 K (5 sets of data were used).

In this section we present the analysis of the 10 30 ns MD simulations, five at 280 K and five at 300 K, whose configurations are saved every 75 fs in order to describe the details of the kinetics during the final nucleation and full growth process. Because of the tremendous size of the resulting simulation data, we concentrated our analysis on a 10 ns window centered around the drop in energy (Figure 7). Panel (a) represents the average energy taken over all five simulations as a function of time at 280 K. Trajectories are aligned, in time, at the point at which they reach −80 kcal/mol, which is roughly the midpoint in the energy drop for all simulations. Most of the energy drop associated with oligomeric growth, on the order of 600 kcal/mol
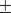
100 kcal/mol, takes place over 4 ns, in agreement with our earlier observations for a typical aggregation process at 280 K. The relatively small error bars along the energy curve indicate the good reproducibility of the properties over time at 280 K. At 300 K, the growth phase associated with the energy drop, of about 450 kcal/mol
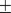
200 kcal/mol, also takes on the order of 4 ns (Figure 7(b)), similar to a 280 K energy drop. The standard deviation on the 300 K curve is, however, greater than at 280 K, demonstrating a greater variability associated with larger thermal fluctuations.

#### Sigmoidal growth and lag time

**Figure 8:**
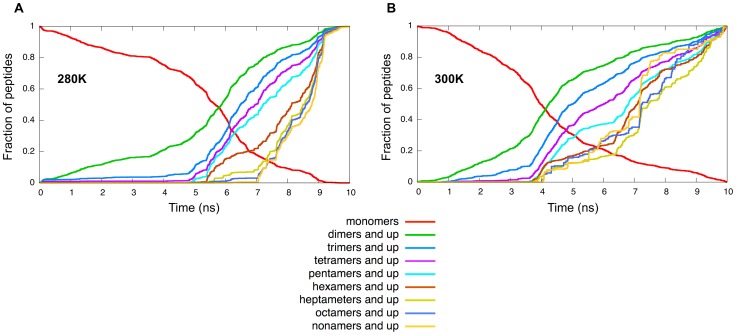
Size evolution. (a) Fraction of peptides occurring as monomers and cumulative curves for all aggregates size up to 9 monomers at 280 K, i.e., probability of finding an aggregate of at least size 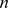 for various 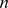. (b) Same as (a) at 300 K. One simulation run is shown at each temperature, for which data was collected for a 10 ns window centered around the energy drop.

In Figure 8 (a) and (b), we plot the cumulative size of various aggregates as a function of time over the 10 ns period, i.e., the occurence probability for oligomers of size at least as large as indicated in the graph. Aggregates with a minimal size larger than one show a sigmoid-like growth starting from about the middle of the energy drop, at 5 ns, with an increasing lag time the bigger the species. Sigmoidal aggregation kinetics have been largely observed experimentally [11], [28], [74]–[81] and numerically [82]–[85] and are a well-established characteristic of a nucleated-growth process. Similar cumulative curves have been obtained for Monte-Carlo simulations of large systems of hexapeptides [85] which indicate that the cooperativity between contacts plays a crucial role in the growth and stabilization of all sizes of aggregates.

#### Origin of the energy drop associated with aggregation

**Figure 9:**
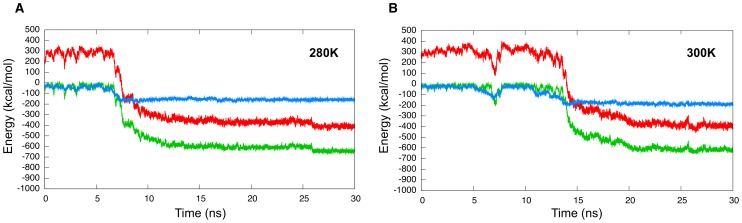
Energy contributions to the total potential energy at (a) 280 K and (b) 300 K. This figure shows one entire 30 ns run for both panels (a) and (b). The energies shown are the hydrophobic/hydrophilic energy (blue curve) and the hydrogen bonding energy (green curve). The total potential energy is shown in red.

Looking at the correlation between the different energy components and the global energy profile at 280 K and 300 K (Figure 9), we observe that the two main contributions to the energy drop come from the hydrogen bonding energy and the hydrophobic/hydrophilic interaction energy. First, the initial collapse of the peptides is accompanied by a decrease in the hydrophobic energy (blue curve, Figure 9) quickly followed by a decrease in the hydrogen bonding energy, which becomes increasingly dominant over the hydrophobic energy, as previously observed in another numerical study of the aggregation kinetics of amyloid peptides [14].

### Nucleus characterization

**Figure 10:**
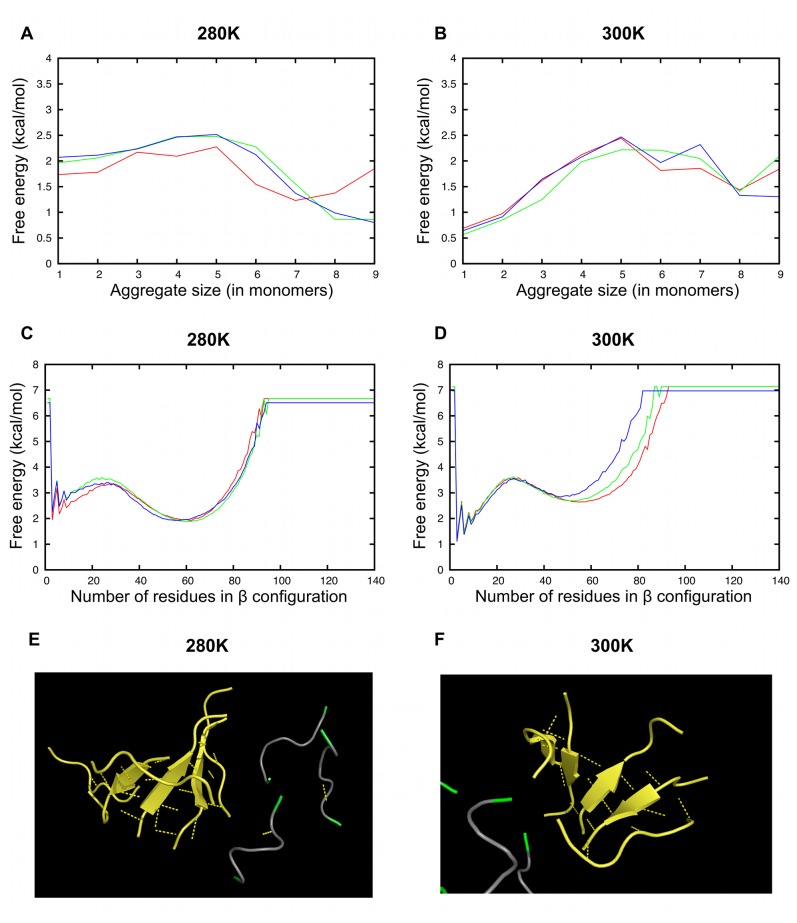
Critical nucleus characterization. (a) Free energy as a function of the aggregate size at 280 K. (b) Same at 300 K. In both (a) and (b), the maximum of free energy corresponds to a critical nucleus size of 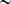
5 monomers. (c) Free energy as a function of the number of residues in a 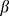 conformation at 280 K. (d) Same at 300 K. In both (c) and (d), the first maximum in free energy represents the critical amount of secondary structure necessary for a nucleus to be stable and trigger aggregation. (e) Typical structure at 280 K of a pentamer nucleus with the critical amount of secondary structure shown in panel (c). (f) Typical structure at 300 K of a pentamer nucleus with the critical amount of secondary structure shown in figure (d).

At both temperatures 280 K and 300 K, aggregation is generally triggered by the formation of a small-sized metastable aggregate, which appears to be stable after a certain lag time. This suggests that we are in the presence of an assembly sequence that can be classified as a nucleated-growth process [24], [26], [84], [86]–[90], i.e., that this small metastable aggregate, which we term nucleus, serves as a nucleation center of the aggregation process. The 152 100 ns MD simulations were divided in 3 sets at both 280 K and 300 K and we computed the free energy as a function of aggregate size and secondary structure for those 3 sets of simulations in order to determine the size and amount of secondary structure of the critical nucleus (Figure 10). Performing this task on different sets of data allows us to have an idea on the order of the fluctuations in the free-energy. At 280 K the nucleus size corresponding to the maximum of free energy is found to be between 4 (Figure 10(a) - green curve) and 5 monomers (Figure 10(a) - red and blue curves) and between 5 (Figure 10(b) - red and blue curves) and 6 monomers (Figure 10(b) - green curve) at 300 K. This result is expected since larger thermal fluctuations require a bigger aggregate to survive and lead to growth. The pentameric critical nucleus identified here is also near the critical size estimated by Nelson *et al.* [33] and by us, in a previous thermodynamic study [39].

As was shown recently [91], [92], the critical nucleus size in a finite-size system is systematically overestimated and it is necessary to correct for this artifact. From the classical nucleation theory (CNT), Grossier *et al.* derive an expression for the total free energy of forming an aggregate of size 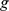 monomers in an infinitely large system to be [91]:
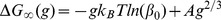
(2)where 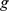 is the aggregate size, 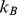 is the Boltzmann constant, 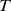 is the temperature, 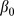 is a dimensionless constant and represents the supersaturation and 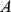 is the interfacial energy (or surface tension) taken to be a constant in the model. Due to our very small system size, a 20-mer, and the low critical nucleus, it is not possible to obtain a good fit to this continuous equation. However, the overestimation correction could explain the slight difference we observe with respect to the experimentally-derived critical nucleus of four monomers.

Looking at the free energy barrier of forming a certain amount of secondary structure, we find that a viable nucleus requires between 24 and 28 residues in 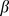 conformation at 280 K while it requires between 27 and 29 residues in 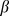 conformation at 300 K (Figure 10 (c) and (d)). The increase in free energy for 80 residues is due to the finite-size effects of our system. It becomes harder to have 80 residues in 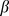-conformation as no more monomers are available to the system to continue growth. Figure 10 (e) and (f) show the dominant pentamer nucleus structure having such amount of secondary structure at 280 K and 300 K. In both cases, the pentamer seed is partially ordered. In most cases, no more than a dimer is formed beside the nucleus.

### Proposed mechanism

To assess the microscopic mechanisms involved in the kinetics, we first identify all types of association and dissociation: growth by monomer addition (and, reversibly, loss by monomer subtraction), growth by fusing two oligomers together (and, reversibly, fragmentation of one oligomer into two smaller oligomers at least 2 monomers in size) and the direct formation/destruction of oligomers from/into monomers. In this section, we refer to any aggregate bigger than one monomer as an oligomer. It is important to point out that there is a wealth of “monomer addition” models for diverse polymer-forming proteins such as actin [93], [94], tubulin [82], the sickle cell hemoglobin [25], [74] and amyloid proteins such as A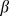 [27], [95], [96], 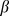
2-microglobulin [79] and Sup35 [78]. There also exists numerous “oligomer fusion” models for A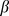 [9]–[11], [97], 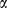-synuclein [73], [97], [98], the phosphoglycerate kinase protein [99], the lysozyme protein [100] and Sup35 [65], [101], some of which have observed both processes happening at the same time.

Association and dissociation rates were calculated, with our clustering code, every 75 fs over a 10 ns window (centered around the energy drop) for the 30 ns simulations and as described in Eq. (1). Then, for each time interval, we calculated the total number of events, originating either from monomer addition/loss, from oligomer fusion/fragmentation or from monomers
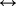
oligomers events across all species such as:
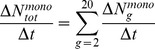
(3)and
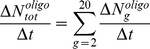
(4)where 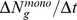 and 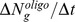 are the “monomer addition/loss” and “oligomer fusion/fragmentation” + monomers
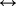
oligomers components of Eq. (1).

**Figure 11:**
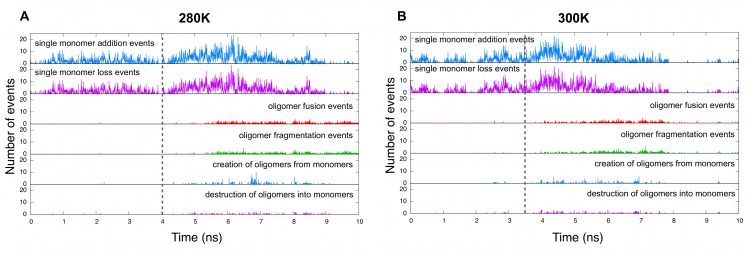
Aggregation mechanism. Time evolution of association and dissociation events before during and after nucleation by either single monomer events (meaning one at a time), by oligomer fusion/fragmentation, or by formation/destruction of oligomers from/into monomers at (a) 280 K and (b) 300 K. The dashed grey line indicates the beginning of nucleation. In (a), at 280 K, the aggregate stops growing in size after t = 9 ns while in (b), at 300 K, the aggregate stops growing just before t = 8 ns. For ease of reading, each point in the graphs is the sum of events in 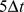 (5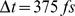).

Figure 11 shows the evolution of these two quantities for both association and dissociation events at 280 K (Figure 11(a)) and at 300 K (Figure 11(b)). We differentiate the fusion/fragmentation events from the formation/destruction of oligomers (bigger than dimers) directly from/into monomers. At both temperatures, the data clearly shows that the assembly mechanism is dominated by “monomer addition/loss” events. Then when nucleation and aggregation happen, we see a notable increase in the amount of monomer events and a trigger of “oligomer fusion/fragmentation” and “monomers
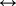
oligomers” events. We notice a well-defined increase in the number of “monomer addition/loss” events just before the first “oligomer fusion/fragmentation” events appear. This increase corresponds to the start of nucleation and suggests that once nucleation is triggered and most of the monomers are recruited, they join different sites, or clusters, that will later on fuse together to form a larger oligomer. Later, when the aggregate stops growing in size, we observe no more “single monomer” or “monomers
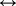
oligomers” events and observe, in some cases, the presence of only fusion and fragmentation of oligomers (Figure 11(a)). This means that further rearrangements in the structure during the stabilization phase are accomplished mainly through oligomer-involving events, if any.

### Conclusion

We presented here a detailed study of the onset of amyloid aggregation for 20-mers of GNNQQNY. Using molecular dynamics with the OPEP coarse-grained force field, we show that nucleation of this small amyloid peptide is dominated by monomer addition/loss events, with very small contributions from larger oligomers, following closely the classical nucleation theory. It is then meaningful to extract a critical nucleus, that can be obtained from the calculation of the free-energy as a function of nucleus size. We find that, at 280 K, this critical size is between 4 and 5 monomers, while it is between 5 and 6 at 300 K, in good agreement with the experimental estimate of 4 monomers [33], especially when taking into account the finite-size bias that tends to overestimate the size of the critical nucleus [91], [92]. Correspondence with CNT stops there, however, as the kinetic process associated with aggregation and growth differs in two majors from this theory. First, while most of the structural organization takes place during the 4 ns growth process, aggregates continue to mature by collective motions, slowly dropping in energy as hydrogen bonds and 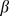-sheet content evolve. Second, nucleation does not lead to a single structure, but shows clear polymorphism with a distribution of assemblies that can be classified into two distinct categories: a compact oligomer made of a number of relatively short 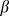-sheets, typically three, and a more extended fibril-compatible two-sheet structure. These structures represent well-separated local basins and the only way to move between them, in our simulations, was through a complete dissociation and reassociation of the monomers. The well-defined polymorphic nature of GNNQQNY is in line with experimental and numerical observations in other amyloid sequences, such as amyloid-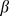. It was shown there that the protein could adopt multiple fibrillar structures [18], [102], but also off-pathway 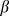-barrel organizations that would be responsible for at least part of the toxicity. [103] For GNNQQNY, the two polymorph families observed here are close enough that they should lead to different fibrillar structures rather than on and off-pathway organizations. Only simulations with a larger number of peptides will be able to tell.

How much of these results can be applied to experimental studies of GNNQQNY? A previous stability study of the structures predicted with OPEP using explicit SPC solvent and all-atom GROMOS96 showed that our simulations are realistic, except for the most extended structures [39]. If the growth time is not directly extendable to all-atom systems, the thermodynamics and, therefore, the critical nucleus size but also the polymorphism, which is a signature of amyloid aggregates, should be valid. Our results suggest that the specific shape, out of a family of structures, is selected very early on and that moving from one to another requires going over a very high barrier, high enough that it was never observed in our simulations, the preferred being going first through a complete dissociation. Such behavior could change with larger aggregates, and the direct rearrangement become more favorable than complete dissociation. Only further work, on larger systems, will show whether new families of structures are possible for GNNQQNY and if the CNT applies when more monomers are in play. Our results on the 20-mer of GNNQQNY are at least compatible with experiments and offer a number of insights into the onset of aggregation and polymorphism for small amyloid peptides.

## Supporting Information

Figure S1
**Characteristics at 300 K for the GNNQQNY 20-mer as a function of the number of hydrogen bonds and of the number of side chain contacts.** (a) Time evolution map of the system. Black regions indicate the beginning of the simulation while yellow regions indicate the end. (b) Density map representing the probability of having a configuration lie in a specific region. Yellow is the highest density and red the lowest. (c) Proportion of parallel 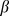-strands. Yellow regions indicate that 100% of the strands are in parallel orientation while black regions indicate that none of the strands are in parallel orientation thus meaning that they all are in antiparallel orientation. In all three plots, the nucleation region is denser, due to bigger thermal fluctuations at 300 K, which destabilize early metastable aggregates.(TIF)
