# Supplementary figures and images for: Kinetics of Amyloid Aggregation: A Study of the GNNQQNY Prion Sequence
Source: PLoS Comput Biol. 2012 Nov 29;8(11):e1002782. doi: 10.1371/journal.pcbi.1002782 (PMC3510058; doi:10.1371/journal.pcbi.1002782)

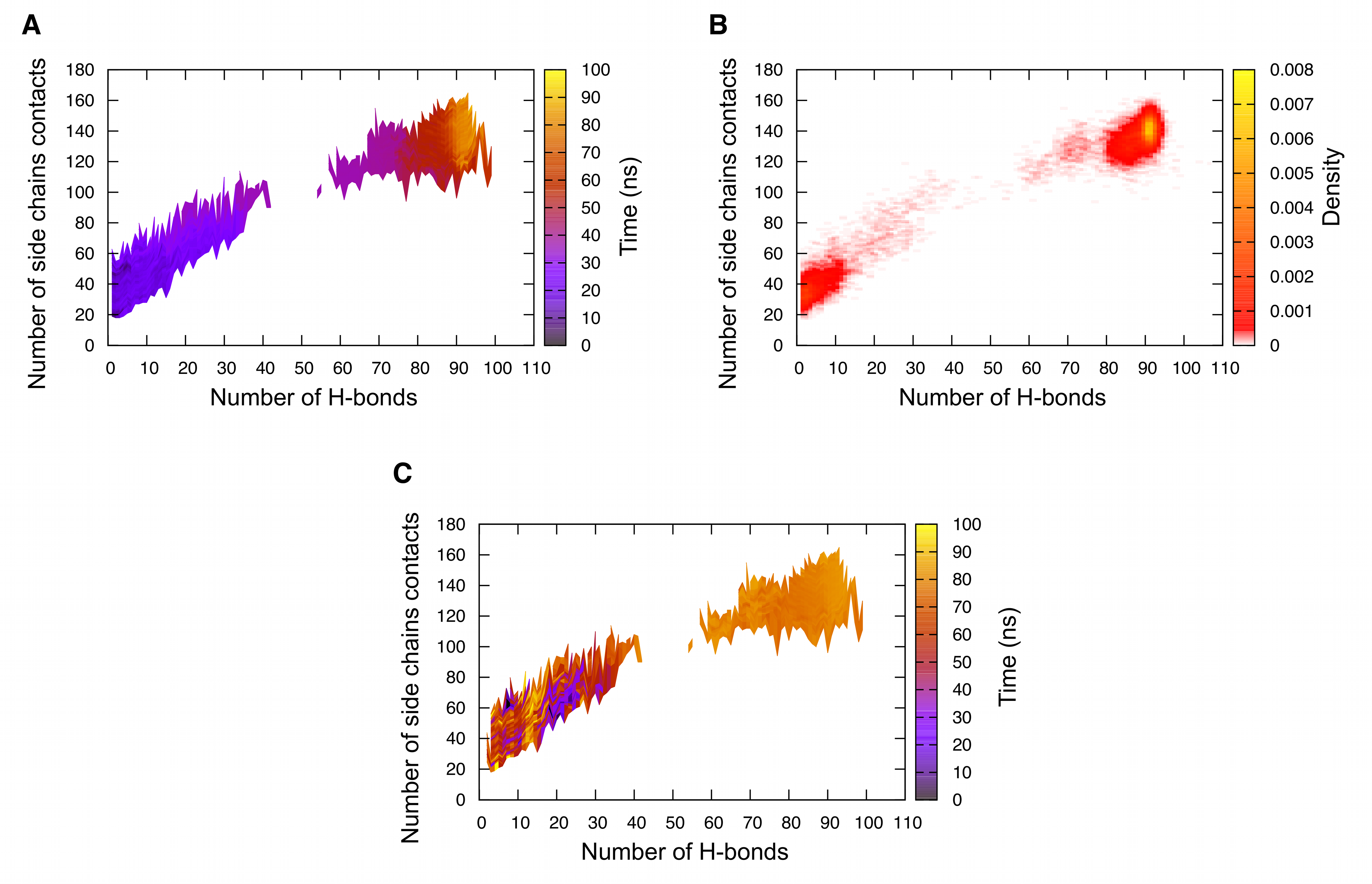

Supplement: Figure S1 — Characteristics at 300 K for the GNNQQNY 20-mer as a function of the number of hydrogen bonds and of the number of side chain contacts. (a) Time evolution map of the system. Black regions indicate the beginning of the simulation while yellow regions indicate the end. (b) Density map representing the probability of having a configuration lie in a specific region. Yellow is the highest density and red the lowest. (c) Proportion of parallel -strands. Yellow regions indicate that 100% of the strands are in parallel orientation while black regions indicate that none of the strands are in parallel orientation thus meaning that they all are in antiparallel orientation. In all three plots, the nucleation region is denser, due to bigger thermal fluctuations at 300 K, which destabilize early metastable aggregates. (TIF) [file pcbi.1002782.s001.tif]
